# Loot

**Published:** 1872-11

**Authors:** 


### Loot.

***Mortality Statistics of the Three Learned Professions.***

Dr. J. M. Toner, Washington, D. C. (*Boston Med. and Surg. Journal*), has compiled the following statements of the number of deaths returned from the Census of 1870, as occurring among the three learned professions of our country—theology, medicine and law—for the year ending June 30, 1870. He has been unable to obtain the number returned as engaged in each of these professions for the year 1870. For the year 1850 there were returned 28,842 clergymen, 40,564 physicians, and 23,939 lawyers. For the year 1860 there were returned 37,529 clergymen, 54,543 physicians, and 33,193 lawyers. It is fair to presume that each retains about the same proportion to each other in the census of 1870. The number of deaths reported among clergymen in all the States and Territories of the United States for 1870 was 629; among physicians, 947; and among lawyers, 595.

| Causes of death assigned. | C. | P. | L. |
|---|---|---|---|
| Unknown, | 10 | 15 | 1 |
| General Diseases, | 242 | 344 | 234 |
| Diseases of the Nervous System, | 77 | 143 | 234 |
| " " Circulatory System, | 54 | 73 | 54 |
| " " Respiratory System, | 84 | 130 | 58 |
| " " Digestive System, | 76 | 105 | 61 |
| " " Urinary and Generative Organs, | 32 | 37 | 19 |
| " " Organs of Locomotion, | 3 | 4 | 0 |
| " " Integumentary System, | 1 | 1 | 3 |
| Condition not necessarily associated with general or local diseases, | 39 | 31 | 19 |
| Poison, | 2 | 18 | 13 |
| Accidents and Injuries, | 9 | 46 | 43 |

—*Med. Record.*

### *The Slaughter of the Innocents.*

Attention is frequently called to the mortality among children in particular seasons or particular localities, but few seem to be aware of the frightfully large per centage of infant mortality year by year, compared with the whole number of deaths of all ages. The mortality tables prepared by Dr. J. M. Toner, of this city, whose careful and intelligent labors in gathering the vital statistics of the country, have been referred to heretofore, present some startling facts in this connection. These tables show the annual mortality in several of the States, and in a number of our large cities, made up from their published reports for the years named, giving the whole number of deaths, and also the number dying under five years of age; the number dying between five and ten years; also, the number dying from cholera infantum, with the per cent. of each class.

The census of the United States for 1850 showed a per centage of 39.76 of deaths of children under 5 years of age to the total deaths of all ages; and the per centage in the census of 1860 had increased to 43.15. The mortality under 5 in the great cities of the country has risen to a much higher rate, the highest being in Chicago, where it has mounted from a per centage of 28.67 in 1843 to 62.83 in 1869. In 1868, the number of deaths of children under 5 to the total deaths of all ages stood in that city in the frightful proportion of 3,713 of the former, to 5,984 of the latter, and in 1869 it had risen to 4,077 under 5 to 6,488 over that age.

Next comes St. Louis, where, in 1871, the per centage was 51.10; the number of deaths under 5 being 3,409 to 6,670 above.

Ranging about the same as St. Louis is the per centage of infant mortality in New York. In New York city in 1869 the per centage was 51.09; the number of deaths under 5 in that year being 12,359 to 25,167 above. In that city it would appear, however, that the proportion of deaths under 5 is rather diminishing than otherwise, as during the series of years from 1835 to 1853 it had ranged from 51.02 to 57.10, while in 1866 it was but 47.73, in 1867 it was 52.10, and in 1869 but 51.09. In the year 1811 the mortality in New York amongst the children seems to have been exceptionally large, as the per centage was 77.09; the number of deaths under 5 being 1,946 to 2,524 above that age. In Philadelphia, too, the chances of the little folks in the fight for life seems to be rather improving, as the per centage of deaths under 5 stood as high as 51.87 in 1853, and 51.25 in 1861, while from 1862 to 1869 it has ranged from 43.22 to 46.63, and in 1870 was but 44.27.

Next to New York in infant mortality comes Baltimore with its per centage, in 1869, of 49.85 under 5 years, though the proportion has increased but little since 1860, when it stood 48.74.

Cincinnati, in 1868, had a per centage of 46.68 under 5; and Philadelphia, as above stated, the next highest per centage. In New Orleans the returns are brought down only to 1857, when the per centage was 43.85. Providence, R. I., seems to be a favorable place for the little ones, as the mortality under 5 has not, from 1865 to 1870, been above 36.99.

In this city the mortality returns have been so inadequately made that only scattered statistics covering separate years can be obtained. In 1849 the number of deaths under 5 was 400, to 866 above; a per centage of 46.18. In 1852 there were 547 deaths under 5, to 1,115 above; a per centage of 49.05. In 1858 the per centage was 45.78, the number of deaths under 5 being 429 to 937 above.

The mortality statistics of Richmond present some curious features, as showing that while the number of deaths of colored children under 5 is nearly two to one that of the whites of the same age, the proportion of white children dying of cholera infantum is nearly double the colored mortality from the same disease, there being 490 white deaths in 1869 of cholera infantum to 250 colored in the same year in that city.

In view of the appalling proportion of infant mortality, especially in our large cities, shown by the statistics above referred to, Dr. Toner some time ago called attention to the necessity for adopting some general measures for checking this vast waste of infant life. We have various humane institutions, insane asylums, etc., to alleviate the sufferings and lengthen out the lives of the wrecks of humanity, but we have literally nothing to stay the immense waste of life of the hopeful young, full of great promise. It is certainly better to provide for the preservation of the lives of those who will grow into usefulness, than to confine all our humane efforts to guarding the health of the hopelessly shattered wrecks.

Of course wealthy parents have it in their power to remove their ailing children to purer air, but it is an unpleasant task for a physician to say to parents, unable to meet the expense, that the life of a child suffering from cholera infantum depends on its being removed to another locality. To meet this difficulty, Dr. Toner suggests the establishment of free parks at elevated points, where parents, or mothers and nurses, may camp out in the summer months in such style as suits their means or convenience, either in tents or cheap cottages. Cities and communities should feel it a moral duty to aid in any measure of the kind for the preservation of infantile life. Cheap fare, or free fare, should be furnished to poor women with their sick babes, and they should be aided with rations on the ground. Philanthropic people no doubt would willingly aid, if the means were pointed out, in some systematic measure of relief for the suffering little ones who perish by the thousands yearly in our large cities, for want of air and attention. The child in such cases is not sick, but is suffering simply from excess of heat. There is no solid tissue in the infant. It is virtually all liquid; it gets heated up and deterioration takes place. What is wanted is to keep down the temperature. Infants do not perspire as adults do; but the process of retained heat goes on with them, and they suffer accordingly. Evaporation should be encouraged; they should be sponged frequently and exposed to the air as much as possible; and, above all, there should be no flannels next to the skin in hot weather. Many mothers really destroy all chances their ailing babes have for life by bandaging them with flannels, and sedulously keeping them out of the fresh air lest they should take cold in a draught. Infants, for the reasons above explained, can hardly have too much air.—*Washington Paper*.

***Treatment of Small-Pox by Baths.***

At a recent meeting of the Dublin College of Physicans, Dr. H. Benson called attention to a form of treatment so prominently brought before the Society on a late occasion by Dr. Stokes. He referred to the treatment by the bath. He was so struck by the result in Dr. Stokes' cases that he determined to adopt the treatment in the next suitable case he met. In a very few days such a case presented itself. The patient was a gentleman residing in one of the suburbs of Dublin. He suffered from an extremely bad form of confluent small-pox. It was remarkably confluent, not only on the face, but also on every part of the body. The pustules were not well filled but were flat, and the face presented the appearance as if a wax candle had been dropped over every part of it. During the secondary form the delirium became exceedingly troublesome, and the patient quite uncontrollable. For the previous twenty-four hours he had not been in bed for five minutes, and he had had no sleep for over thirty-six hours. Hypnotic remedies had no effect, and it was not possible to apply leeches or other applications to the head. With some difficulty he was placed in a slipper-bath, of the temperature of 98°, and he immediately exclaimed, "it's glorious, it's delicious, it's delightful." He became at once calm, collected, and obedient, and within fifteen minutes he ceased to have any delirium. After half an hour he slept in the bath for two hours, occasionally waking for a minute or two while fresh water was being added. He (Dr. Hawtrey Benson) kept the patient in the bath for five hours and a half, removing him after that on account of headache which supervened. He was then put to bed, perfectly free from delirium, and with the help of fifteen grains of chloral (of which four times that dose had no effect previously) he slept uninterruptedly for eight hours. The case progressed from that out without the slightest check. Dr. Benson thought that this treatment did not receive its due measure of attention at the hands of the profession.

Dr. Grimshaw asked, what was the temperature of the man's body when he went into the bath?

Dr. Benson replied, that the temperature could not have been lower than 104°, and that the bath was at least six degrees lower.

### *Citrate of Iron and Bismuth a New Remedy for Dyspepsia.*

By Charles Rice.

Although I call this preparation new, it has been in use for several years in the public hospitals and dispensaries of this city, and also in private practice, and has acquired the reputation of being one of the most prompt and valuable remedies at present known for gastric disturbances, depending upon an abnormal or defective digestion generally, and particularly so for the gastric intolerance of consumptive patients. Its action is often so prompt that one full dose has in many instances afforded immediate relief.

Being requested some years ago to devise a liquid preparation containing Bismuth and Iron (at that time intended for use in some other complaints), I finally, after various trials, adopted the following formula, which I have followed ever since:

Take of Citrate of Bismuth, Ammonio-Citrate of Iron, each 320 grs.; Water of Ammonia, Water, each a sufficient quantity.

With four ounces of Water rub the Citrate of Bismuth into a smooth paste; gradually add Water of Ammonia until solution takes place, being very careful not to have an excess of Ammonia. Now add the Ammonio-Citrate of Iron and some more water; dissolve, filter, and wash the filter with enough water to make the solution measure one pint.

This solution, if intended to be long kept, may be partly made up with Glycerin, although I cannot speak from experience whether it is so well borne by the stomach. A more useful addition, however, is good sherry wine, of which there may be used ten fluid ounces, (or perhaps more), in place of so much water.

The above solution is prescribed under the name of Liquor Ferri et Bismuthi Citratis, and contains in one fluid-drachm two and a half grains each of Citrate of Bismuth and Ammonio-Citrate of Iron. The dose is from one to two fluid-drachms, half an hour before meals, or—when required—after meals.

It is, of course, no true double salt, chemically speaking, but only a mixture of Ammonio-Citrate of Bismuth and Ammonio-Citrate of Iron; and, although a true double salt containing those elements might perhaps be prepared, I doubt whether it could have any better effects.

The solution may also be prepared of a concentrated state, and spread upon plates of glass to dry, yielding exceedingly handsome scales of a golden-brown color, which must be protected from the light, and five grains of which are equal to one fluid-drachm of the solution.—*American Journal of Pharmacy.*

### *Phosphorus in Skin Diseases.*

This has been highly lauded by Dr. Fox and others. In an article in the *Dublin Journal of Medical Science*, Dr. Eames says in reference to its continued use:

I have found that phosphorus produces a coated state of the tongue, not unlike the silvery tongue which follows the prolonged use of arsenic. Loss of appetite, mental depression, and bodily weakness, also are induced much earlier in some cases than in others, but to be watched for in all cases in which the drug is given. On the earliest appearance of dyspeptic symptoms I now discontinue its use, and administer some of the mineral acids. Many patients have mentioned that some two or three minutes after taking the medicine a pleasant sensation of warmth is felt through the entire body. I have not ascertained that any aphrodisiac effect is produced, though I have frequently inquired after it. A slight diaphoresis is observed occasionally. Diarrhœa was not induced in any of my cases. The amount of urine in some was slightly increased. An analysis made by my friend Dr. Cameron failed to detect any deviation from the standard of health.

Most of the foregoing cases had been treated by arsenic and other drugs before coming under my care. I have at hand notes of numerous other cases, which have been from the first treated successfully with phosphorated oil; these I have not detailed, as the intention of the present paper is to prove that the administration of phosphorus will cure certain cases of cutaneous disease, even after mercury, arsenic, and other remedies, vaunted as specifics, have completely failed to do so. I may mention, however, that eczema of the scalp, with enlargement of the glands of the neck, is very amenable to treatment by phosphorous. The strumous character of this disorder, and the difficulty of its removal, are very generally admitted. I have seen in many cases a copious eczematous eruption on the head and behind the ears, combined with greatly enlarged *glandulæ concatenatæ*, disappear in a few weeks after this treatment had been adopted, the glands being also quickly restored to their normal size.

***On the use of Pepsine Wine in the artificial Feeding of Infants.*** By W. J. Cummins, M.D.

* * * * There is nothing, of course, like a good breast of milk for an infant, if it can be had; and in "the good old times," when the peasantry and small farmers lived on potatoes and milk, without stimulating their nerves with strong tea, nor their brains with penny-a-liner's words, there was an ample field for the selection of a foster-parent; but now even when the *rara avis*, a good nurse, is procured, she is so independent and knows her power so well, that any caprice must be humored, and she is always ready to throw up her situation or neglect her charge. A wet-nurse is, then, an admitted torment, and a balance struck between its advantage and disadvantage is generally against the former.

Artificial feeding by bottle is a great improvement upon the old system of spoon-feeding, as the act of suckling stimulates the salivary glands and insures due in-salivation, which is an important part of infantile digestion. With such an aid the stomach of most *human* infants is vigorous enough to fall into the way of digesting *cow's* milk, properly diluted, and mixed with sugar and cream to assimilate the proportions of its constituents to human milk—but besides the relative excess of casein and albumen contained in cow's milk when compared with human, the coagulum of the latter is "soft, flocculent, and not so thoroughly separated from the other elements of the fluid as the firm, hard curd of cow's milk is from the whey in which it floats." (West.)

When we reflect that the digestive organs of the *human* infant are found to digest human milk, and the force of its gastric juice proportioned to the solution of its soft, flocculent coagulum, we can understand why the solvent power of its gastric juice is sometimes unequal to redigesting the firm curd of cow's milk. When such is the case, acetous fermentation is quickly set up; offensive gases distend the stomach and taint the breath, vomiting and diarrhœa set in, and in process of time the little patient sinks into a miserable state of marasmus, and dies. The remedy for this state of things is simple, for although we cannot change the elementary composition of the milk we have to use, we can introduce into the infant's stomach a digestive power proportioned to the food it has to use—the organic principle of digestion taken from the stomach of the calf.

It is now many years since I first applied this simple theory to practice in the case of one of my own children, who, when about three or four months old, was reduced to a condition of marasmus by vomiting and diarrhœa due to imperfect digestion of cow's milk. I ordered him fifteen or twenty drops of Pepsine wine, to be given immediately before or after each meal. Soon after commencing it he began to improve, and by degrees all bad symptoms vanished, and nutrition was quite restored.

The Pepsine was continued until he was nearly two years old, and he throve at least as well as if he had been wet-nursed; other treatment, of course, pre-aided and accompanied the use of Pepsine, but it was not until the latter was commenced that improvement took place.

Shortly after, a child born in England, and bottle-fed, was brought over to this country when about six months old; he also was suffering from infantile dyspepsia, and was pining away in a listless, apathetic state, quite indifferent to surrounding objects, and appearing as if he would lapse into idiocy from mal-nutrition of the nervous centers. I immediately ordered him Pepsine wine, which produced such beneficial effects, that after it had been continued about twelve months, he had become a bright, intelligent, well-nourished child.

Since then I have never recommended a wet-nurse, and have used Pepsine wine largely in dispensary, hospital and private practice, and have seen many apparently hopeless cases recover under its use.—*Dublin Journal of Medical Science, Feb.*

### *Confession no Proof of Guilt.*

The *Lyon Medical*, of April 28, 1872, refers to the case of a girl, aged twenty, supposed to be seven months pregnant. After an attack of hemorrhage, her size seemed to have considerably diminished; and the girl, being closely questioned on the subject, said that, becoming aware of the discharge, she repaired to the closet, where she stayed ten minutes. She added that all had escaped, but that she had not time to look, as she was being called by her mistress. A midwife and the parish surgeon both declared that the girl had been recently confined. She was now again assailed with questions, and told that, for her own sake, she had better make a clean breast of it, as no fœtus had been found in the closet. Perhaps, it was suggested, she had thrown it into the pigsty. The poor creature at first denied such a thing, but at last confessed that it was so. A search was made, but no child was discovered.

She was tried for concealment of birth, on her own confession, and sentenced to six months' imprisonment. The girl had not been taken into custody in consideration of her free confession, and she quietly proceeded to the jail. When admitted, it was found that she was far advanced in pregnancy, and soon gave birth to a healthy girl. By the French law she could no longer appeal, as more than ten days had elapsed since the verdict; but the judge, having the power of appealing within two months, did so, and the girl was acquitted.

This case shows that confession, which is looked upon as the clearest proof of guilt, cannot always be relied upon. And what shall we say of the surgeon and the midwife? The examination was probably hurried and incomplete, and the conclusion arrived at on seeing the signs of recent abundant hemorrhage. This case, even in a simple obstetrical point of view, is full of valuable hints. —*Lancet, May* 18, 1872.

### *Removing a Foreign Body from the Nose.*

Accidentally opening an old number of "Ranking's Abstract," I read an article headed, "A Novel Mode of Removing a Foreign Body from the Nose," in which is related the case of a child from whose nose surgeons failed to remove a cherry-stone, and were outdone by the village barber, who administered an emetic, and, at the moment when vomiting was about to commence, clapped a handkerchief tightly over the mouth of the child. I was reminded of the source from which was obtained a procedure I have invariably instituted in such cases, and never without success. Very many years ago, that best of practitioners, Dr. J. P. Evans (then residing in Arkansas), when on a visit to his native place, Tazewell, Tennessee, was called to the country to see a child with a foreign substance in its nostril, which had held its position in spite of efforts for its removal directed by the professional skill of "all the region round." On the way, the Doctor was saluted by an aged negro woman, who asked him if he was going to see that child. On receiving an affirmative answer, she said: "Put yer finger 'long side the nose, tother side from the thing, and with yer own mouf over the child's mouf, blow hard, and its bound to come out." He followed her directions, and occasioned the result as she had predicted. R.—*Atlanta Medical and Surgical Journal.*

### *Styptic Cotton.*

Dr. Rohland, of New York (*The Medical Record*) has prepared a styptic cotton (*Gossypium Stypticum*) that promises to be a useful addition to the means of arresting passive hemorrhages from extensive surfaces. It has the advantage of cleanliness and convenience, and possesses no irritating properties. It is prepared by boiling the cotton in a solution of alum and gum of benzoin, and, after the cotton is dried and picked, it is saturated by perchloride of iron. It is put up in boxes of convenient size.—*Ibid.*

***Inoculation of Tuberculous Matter in the Human Subject.***

Messrs. Paraskeva and Zallonis, of Syra, in Greece, have published in the *Gazette Medical de Paris*, April 27, 1872, an account of five experiments on rabbits, wherein tuberculous matter, either mixed with the food or inoculated, excited deposits of the same kind in the lungs, thus confirming Villemin's investigations. The authors attempted besides a bolder experiment, and inoculated a fisherman of fifty-five years of age with tuberculous matter on the upper part of the thigh. This man was suffering from gangrene of the great toe, in consequence of obliteration of the femoral artery. He steadily refused amputation, and the authors considered themselves justified in undertaking the experiment. The patient died thirty-seven days after the inoculation, and had never been ill before in his life. Seventeen tubercles in the first stage of development were discovered in the apex of the right lung; two were of the size of split peas, and the others as large as mustard seeds. Two more tubercles were observed in the apex of the left lung. The liver looked healthy, but presented two tubercles on its convex surface, of the size of peas. The authors conclude that they have proved their point, but it should be recollected that, in ordinary autopsies, tubercles are often found when their existence from the history could hardly have been suspected. As for animals, it may always be asked whether we can, in all cases, conclude that the phenomena observed upon them would be the same upon man.—*Lancet, June* 8, 1872.

***Erysipelas.***

Professor Broca has lately recommended a fresh plan of treatment, which, according to *L'Abeille Medicale*, he has often successfully employed at the commencement of the disease. This plan is to apply a layer of collodion upon the skin above the part attacked. The layer of collodion, which is to be on sound skin, should be from six to eight centimetres wide, and form a complete circle, separating the healthy skin from that attacked. A slight circular compression is thus produced, and it is rare for the disease to cross this barrier, behind which it speedily fades. The part should be examined once or twice a day, in order at once to repair any fissures, and the collodion should be quite pure, without any oil, which is sometimes added to it.—*The Doctor.*

[A very old plan revived. Ed.]

